# Dose Tapering of Advanced Therapies in Psoriatic Arthritis: Clinical Predictors and Outcomes in a Biosimilar-Dominant Real-Life Cohort

**DOI:** 10.3390/jcm14124099

**Published:** 2025-06-10

**Authors:** Marta Loredo, Estefanía Pardo, Ignacio Braña, Stefanie Burger, Valentina Chiminazzo, Rubén Queiro

**Affiliations:** 1 Rheumatology Division, Central University Hospital of Asturias, 33011 Oviedo, Spain; mloredomart@gmail.com (M.L.); estefaniapardoc@gmail.com (E.P.); i.brana.abascal@hotmail.es (I.B.); stefanie.nam@gmail.com (S.B.); 2 Biostatistics Platform, Health Research Institute of the Principality of Asturias (ISPA), 33011 Oviedo, Spain; bioestadistica@ispasturias.es; 3 Department of Medicine, Oviedo University School of Medicine, 33006 Oviedo, Spain; 4 Translational Immunology Division, Health Research Institute of the Principality of Asturias (ISPA), 33011 Oviedo, Spain

**Keywords:** psoriatic arthritis, biosimilars, TNF inhibitors, tapering, dose optimization

## Abstract

**Background:** Dose tapering in patients with psoriatic arthritis (PsA) who achieve sustained treatment targets is a common but underexplored strategy, particularly in those receiving TNFα inhibitor biosimilars (TNFibs). This study aimed to assess the prevalence of dose optimization and identify factors associated with its implementation in clinical practice. **Methods:** We systematically selected 130 PsA patients with sustained treatment response from a database of individuals treated with advanced therapies. We evaluated the prevalence of dose optimization (defined as sustained dose reduction) and explored associated factors using multivariate logistic regression models. **Results:** Of the 130 patients, 95 were receiving TNF inhibitors and 35 other advanced therapies. Among those on TNFis, 88 (93%) were treated with TNFibs. A total of 32 patients (24.6%) were undergoing dose optimization, including 30 from the TNFi group (*p* = 0.002). We found that 7 of the 88 patients on TNFibs (8%) experienced loss of therapeutic response during follow-up. One in three patients on TNFis underwent dose tapering. Factors independently associated with dose reduction included no history of tobacco exposure [OR 3.98, 95%CI: 1.3–14.2; *p* = 0.021], male sex [OR 3.26, 95%CI: 1.26–9.04; *p* = 0.018] and use of TNFis as first-line advanced therapy [OR 4.8, 95%CI: 1.7–16.7; *p* = 0.003]. **Conclusions:** Approximately one in four PsA patients who achieve sustained treatment targets undergo dose optimization, most commonly with TNFibs. This strategy appears to be more feasible in male patients, non-smokers and those treated with TNFis as a first-line option.

## 1. Introduction

The therapeutic offer for patients with psoriatic arthritis (PsA) has expanded significantly in recent years with the arrival of new biological (b) and targeted-synthetic (ts) disease-modifying antirheumatic drugs (DMARDs). The positioning of these novel therapies is not entirely clear, although they all have compelling evidence to support their use in everyday clinical practice [1]. Among the novelties that have appeared in recent years in the therapeutic field of immune-mediated inflammatory diseases such as psoriatic arthritis (PsA), rheumatoid arthritis (RA), psoriasis or axial spondyloarthritis (axSpA), the advent of biosimilars of some of the most classic bDMARDs, such as TNFα inhibitors (TNFis), stands out. Biosimilar therapies are not exact copies of the reference molecule, but they do offer sufficient pharmacodynamic and pharmacokinetic similarities to be successfully used in the same indications as their reference counterparts [2]. Currently, although there are no direct clinical trials conducted with biosimilars in PsA, all biosimilars of TNFis whose original molecules had an indication for treating PsA are successfully used in clinical practice [2].

Another relatively new aspect in the management of PsA is the approach of reducing, or even discontinuing, high-impact therapies once treatment goals (remission or low disease activity) have been achieved in a sustained manner [3]. Until recently, this practice was based on case series or small observational studies, but in the last few years randomized controlled trials have been carried out that advocate a more widespread use of this type of intervention [3,4,5]. However, the European Alliance of Associations for Rheumatology (EULAR) advocates a reduction in dosage rather than a suspension, given the high probability of reactivation of the disease in the latter case [6]. Reducing the dose of biological therapy indeed means reducing the dose administered in each drug exposure, or increasing the dose interval, although it is not yet known which would be the best option. In any case, what is known is that, although patients undergoing dose tapering have a much lower serum concentration of the active principle, the sustainability of remission or low activity is maintained with similar efficacy to when serum concentrations are much higher, which is what occurs when the standard dosage of these therapies is used [7]. Thus, when the indication is appropriate, dose reduction of biological therapies allows cost savings, without compromising the maintenance of therapeutic objectives, and with a potential reduction in the incidence of adverse events. Therefore, rheumatologists currently have a double possibility of optimizing their interventions from a cost-effectiveness point of view; on the one hand, they can increase the prescription of biosimilars; on the other, they can establish dose reduction strategies. Both approaches are certainly desirable, especially for publicly funded healthcare systems, but information on tapering strategies comes from studies with reference medicines, while similar information from studies with biosimilars is virtually absent. In this study, we aimed to analyze the prevalence of dose optimization in PsA patients who had achieved their treatment goal in a sustained manner. We also analyzed the factors that may allow this dose reduction strategy.

## 2. Materials and Methods

### 2.1. Study Design and Ethical Considerations

This retrospective observational study included patients with psoriatic arthritis (PsA) who had achieved sustained treatment targets—defined as clinical remission or low disease activity—over time. Patients were selected through systematic sampling, whereby one in every three patients who met the sustained treatment criteria and regularly attended a PsA clinic at a regional reference center were recruited. The selection period spanned from January to June 2023. A substantial proportion of the selected patients were treated with TNF inhibitor biosimilars (TNFibs). All patients treated with biosimilars had previously been treated with their corresponding reference molecules. Eligibility for switching from the reference biologic to a TNFib required that patients had maintained remission or low disease activity for at least 12 consecutive months. This switching strategy aligns with the sustainability objectives of the public healthcare system of an autonomous region in northwestern Spain and is compliant with both national and regional legislation. All patients were managed within Spain’s publicly funded universal healthcare system, where access to biologic therapies is regulated by standardized protocols, ensuring equitable treatment regardless of socioeconomic status or insurance coverage. For the indication and monitoring of the dose reduction, we followed the recommendations of the Spanish Rheumatology Society and Hospital Pharmacy Society Consensus on recommendations for biologics optimization in patients with RA, ankylosing spondylitis and PsA [8]. Confidence in biosimilar medicines has recently been reinforced by EU experts on biosimilar medicines (Biosimilar Medicines Working Party, or BMWP) and the Heads of Medicines Agencies (HMA) Biosimilar Working Group who have drafted a joint statement explaining the rationale for considering biosimilars approved in the EU as interchangeable from a scientific perspective. This statement has been endorsed by the Committee for Medicinal Products for Human Use (CHMP) and the Biologics Working Party (BWP), 21 April 2023 EMA/627319/2022. In any case, patients were informed and educated individually about what switching from an original biological therapy to a biosimilar represents, and they only participated in this study after accepting the change and giving their written informed consent. This inception cohort—defined by the achievement of sustained treatment goals—formed part of a broader national initiative, the “Spanish Registry of Patients with Psoriatic Arthritis Treated with Biologic and Small Molecule Therapies.” All study procedures related to the registry were approved by the Ethics Committee of the Principality of Asturias (Approval No. 248/19). The study was conducted in accordance with the principles of the Declaration of Helsinki and adhered to the guidelines on biosimilar use in immune-mediated diseases established by the Spanish Society of Rheumatology [9].

### 2.2. Study Variables

Data collection included sociodemographic, anthropometric and lifestyle information, family history of disease, clinical characteristics, laboratory tests, therapeutic aspects, comorbidities and outcome measures. The disease activity score for PsA (DAPSA) was used to estimate disease activity and treatment goals with its standard thresholds for remission (0–4), low activity (5–14), moderate activity (15–28) and high activity (>28). Patients were considered to be on a sustained treatment target if this was indicated by their treating physician and/or if they maintained DAPSA remission/low activity for at least 6–12 months. For the purposes of this study, the dose indicated in the summary of product characteristics was initially reduced by 20–50%, by either reducing the initial dose or increasing the interval between doses [8]. Aside from etanercept, the only way to reduce the dose in cases of s.c. administration is by increasing the dosing interval. Conversely, the dose of drugs administered intravenously may be lowered either by reducing the amount administered in each infusion or by reducing the frequency of infusions. The choice of one or another optimization method was at the discretion of the treating rheumatologist after the patient’s consent.

### 2.3. Statistical Methodology

Descriptive statistics were used to summarize study variables. Categorical variables were reported as frequencies and percentages, while continuous variables were presented as means with standard deviations or medians with interquartile ranges, depending on data distribution. Normality was assessed using the Shapiro–Wilk test. Comparisons between groups (e.g., by sex or drug exposure) were performed using Student’s *t*-test or the Mann–Whitney U test for continuous variables and the Chi-square test or Fisher’s exact test for categorical variables, as appropriate. To explore factors associated with dose reduction, univariable and multivariable logistic regression models were applied. To address potential confounding due to differences in baseline characteristics, we included covariates with clinical relevance or *p* < 0.20 in univariable analysis in our multivariable logistic regression models. Covariates considered for adjustment included sex, age, type of treatment and disease duration. Results are presented as odds ratios (ORs) with 95% confidence intervals (CIs) and associated *p*-values. Although no formal sample size calculation was performed a priori, the sample of 130 PsA patients was deemed adequate for this exploratory real-world study. This number is consistent with other studies evaluating tapering strategies in psoriatic disease. For example, Michielsens et al. included 122 patients in a randomized trial assessing tapering in PsA and axSpA [4]. Furthermore, simulation studies suggest that logistic regression models can be reliably estimated with 10–15 events per variable (EPV); given the 32 patients on dose tapering in our cohort, we were able to explore up to three to four predictor variables in multivariable models with reasonable statistical power and precision. Our findings are thus expected to provide meaningful, hypothesis-generating insights into predictors of dose optimization in PsA. Statistical significance was set at *p* < 0.05. All analyses were performed using R software (version 4.3.1 “Beagle Scouts”).

## 3. Results

### 3.1. Summary of Study Population

The study sample included 130 patients, 66 (50.8%) women and 64 (49.2%) men, with a mean age of 55.6 ± 11.2 years and median disease duration of 8.0 [3.0–13.0] years. All patients were receiving b/tsDMARDs with/without conventional DMARDs. The disease was optimally controlled in most cases with a median overall assessment of disease activity by the treating physician of 2.0 [0.0–4.0], while 112 (86.2%) were in remission/low activity status according to the DAPSA score. Of the total number of patients undergoing TNFis (*n*: 95), 88 (93%) were receiving TNFibs. We found that 7 of the 88 (8%) patients on TNFibs lost therapeutic response after switching (4 due to skin worsening, 2 due to joint worsening and 1 due to worsening of both domains) and were returned to the original biomolecule. The remaining 35 patients were on other advanced therapies. Of the 130 patients, 32 (24.6%) were on sustained dose reduction. Table 1 summarizes the characteristics of the study population.

### 3.2. Differences Based on Sex

The average weight was higher in men (82.6 ± 14.1 kg) than in women (73.1 ± 15.1 kg), *p* = 0.001. More men (30.2%) than women (4.5%) were found to be regular alcohol consumers, *p* < 0.001. More men than women had elevated lipid levels (47.6% vs. 28.8%, *p* = 0.043) and uric acid levels (46% vs. 13.6%, *p* < 0.001). However, average C-reactive protein (CRP) values were higher among women [median 2.50 (0.63–7.05) mg/L vs. 1.20 (0–3.80) mg/L, *p* = 0.017]. Median DAPSA was higher among women [6.17 (2.03–11.1) vs. 4.0 (0.12–7.29), *p* = 0.012]. However, DAPSA remission/low activity rates were not statistically different between men (92.1%) and women (80.3%), *p* = 0.094. More men (84.1%) than women (63.6%) were on TNFis, *p* = 0.015. Also, more men (33.3%) than women (15.2%) were on sustained dose reduction, *p* = 0.027.

### 3.3. Differences Based on Drug Exposure

At study entry, patients on TNFis were younger (54.2 ± 11.5 years) than those on non-TNFi drugs (59.4 ± 9.7 years), *p* = 0.03. In the non-TNFi group, women predominated (70.6%), while in the TNFi group, there were more men (55.8%), *p* = 0.015. More patients in the non-TNFi arm were affected by enthesitis (44.1%) compared to the TNFi group (23.4%), *p* = 0.039. The median CRP value was also significantly higher among patients in the non-TNFi group [4.25 (0.73–8.30) mg/L vs. 1.40 (0–3.65) mg/L, *p* = 0.004]. A significantly higher percentage of patients in the TNFi group (92.6%) were in remission/low DAPSA activity compared with the non-TNFi group (67.6%), *p* = 0.001. More patients on TNFis were receiving concomitant methotrexate (45.3%) than in the non-TNFi group (11.8%), *p* = 0.001. However, more patients in the non-TNFi arm were on leflunomide (20.6% vs. 5.3%, *p* = 0.014). The percentages of patients in first-line therapy were also very different between groups (TNFi: 71.6%; non-TNFi: 26.5%, *p* < 0.001). The vast majority of patients on dose optimization (30 of 32) were in the TNFi group, *p* = 0.002.

### 3.4. Dose Reduction-Associated Factors

**Figure 1:**
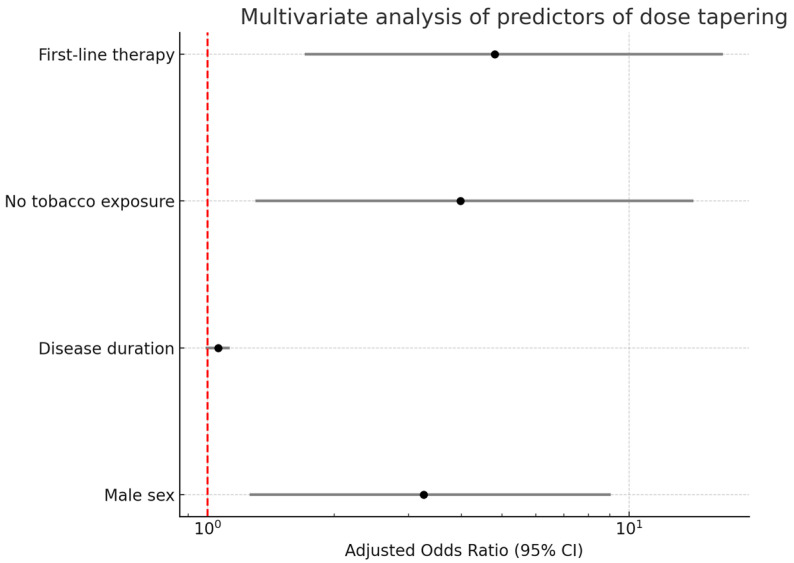
Multivariate logistic regression analysis of factors independently associated with dose tapering in patients with psoriatic arthritis. Adjusted odds ratios (ORs) and 95% confidence intervals (CIs) are plotted on a logarithmic scale. Variables included in the final model were male sex, disease duration, no tobacco exposure and first-line use of TNF inhibitors. The vertical red dashed line represents the null value (OR = 1).

To address potential confounding due to differences in baseline characteristics, we included covariates with clinical relevance or *p* < 0.20 in univariable analysis in our multivariable logistic regression models. In the univariate regression, the factors associated with dose reduction were male sex (OR 2.8, *p* = 0.018), disease duration (OR 1.08, *p* = 0.008), no tobacco exposure (OR 3.94, *p* = 0.012), physician’s overall disease activity assessment (OR 0.54, *p* < 0.001), DAPSA (OR 0.73, *p* < 0.001), TNFi use (OR 15.2, *p* = 0.009) and first-line therapy (OR 4.8, *p* = 0.003). After adjustment for confounding variables, in multivariate regression analysis the factors that were independently associated with dose reduction status were as follows: no tobacco exposure [OR 3.98 (95%CI: 1.3–14.2), *p* = 0.021], male sex [OR 3.26 (95%CI: 1.26–9.04), *p* = 0.018] and first-line therapy [OR 4.8 (95%CI: 1.7–16.7), *p* = 0.003]. Figure 1 shows the forest plot of the multivariate regression. Table 2 shows the full regression models.

**Figure 2:**
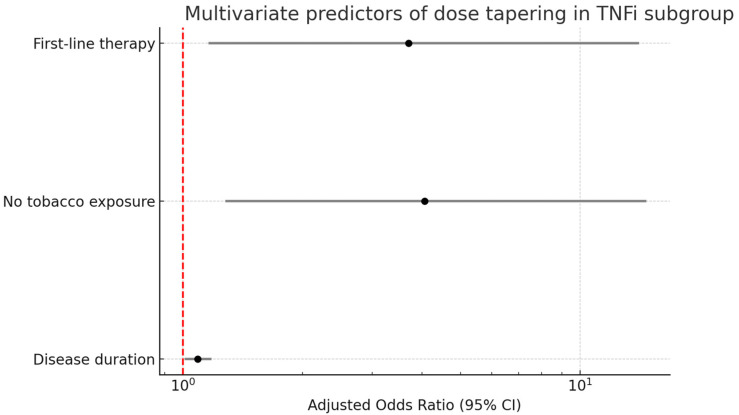
Multivariate logistic regression analysis of predictors of dose tapering in patients treated with TNF inhibitors. Adjusted odds ratios (ORs) and 95% confidence intervals (CIs) are shown for disease duration, no history of tobacco exposure and first-line therapy. Values are presented on a logarithmic scale. The red dashed line represents the null effect (OR = 1).

We then performed a dose optimization sub-analysis in the population exposed to TNFis (*n*: 95), since it was the majority population in this study. In the univariate regression analysis, factors associated with dose optimization were disease duration (OR 1.12, *p* = 0.003), no tobacco exposure (OR 4.1, *p* = 0.015), physician’s global disease assessment (OR 0.57, *p* < 0.001), DAPSA (OR 0.73, *p* < 0.001) and leflunomide use (OR 9.8, *p* = 0.045). The variables independently associated with dose reduction in the multivariate regression model were disease duration [OR 1.09 (95%CI: 1.01–1.18), *p* = 0.030], no tobacco exposure [OR 4.06 (95%CI: 1.28–14.72), *p* = 0.023] and first-line therapy [OR 3.7 (95%CI: 1.2–14.1), *p* = 0.037]. Figure 2 shows the forest plot of the multivariate regression. Table 3 shows the full regression models for the TNFi group.

## 4. Discussion

Our exploratory study yields several interesting results for clinical practice. Firstly, most patients who switched from an original biomolecule to a biosimilar maintained the response achieved by the former. Secondly, almost 1 in 4 patients on treatment targets were potential candidates for dose optimization. Third, almost a third of patients on TNFibs achieved a sustained dose reduction. Finally, male patients, non-smokers and those using a first-line therapy were those who independently associated a higher possibility of dose optimization.

Switching between reference molecules and their biosimilars has no impact on efficacy, safety and immunogenicity in most cases in patients with immune-mediated inflammatory diseases [2,10]. In addition, biosimilars also significantly help improve patient access to biological therapies and contribute to healthcare system sustainability [2,3]. For this reason, the use of biosimilar drugs has become an increasingly widespread practice, especially within those healthcare systems subject to public funding [2,9]. Initial fears about a potential loss of therapeutic efficacy when switching from a bio-original to a biosimilar molecule have been dispelled as more and more studies have been published in this regard [2,3,4,5]. Our study is yet another that confirms that in most patients in whom this switch is made, the therapeutic response achieved with the reference drug is maintained over time with its biosimilar. In fact, in our study only 8% of patients on biosimilars needed to return to the original biological therapy. In addition, the lack of a standardized tapering protocol reflects the real-world clinical practice and may affect internal validity; however, it also enhances the ecological relevance of our findings.

According to the latest EULAR recommendations for the management of PsA, in patients in sustained remission, tapering of DMARDs may be considered [6]. Therefore, from a safety and a cost perspective, drug tapering is a logical step when patients are doing well over time. Until recently, this recommendation was supported by case series and small observational studies, but in recent years well-designed clinical trials have provided greater support to this type of practice. Michielsens et al. performed a pragmatic open-label, monocentric, randomized controlled non-inferiority trial on T2T tapering of TNFis. Among 122 patients (64 PsA and 58 axSpA) randomized to a T2T strategy with (*n* = 81) or without tapering (*n* = 41), the proportion of patients sustaining low activity at 12 months was 69% for the tapering and 73% for the no-tapering group, thus concluding that a T2T TNFi strategy with tapering attempt is non-inferior to a T2T strategy without tapering [4]. In another 18-month, open-label, randomized controlled trial including 160 patients treated with etanercept, tapering could be carried out without losing efficacy in RA, PsA and axSpA patients in sustained low activity. A substantial proportion of patients could stop etanercept for at least 6 months and low drug concentrations proved sufficient to control disease activity [5]. Despite accumulating evidence on dose tapering strategies in immune-mediated diseases, in a recent systematic literature review with meta-analysis, the strategy of TNFi tapering was associated with a significantly increased risk of disease flare (OR 1.60) compared to maintaining SpA (including PsA) patients at the standard TNFi dose [11]. Thus, further studies are still needed to determine which patients can safely undergo the tapering of TNFis and to develop safe tapering strategies.

Factors reported to be associated with an increased likelihood of maintaining remission on a tapered biologic dose include lower disease activity and longer duration on TNFis prior to tapering [3]. However, concomitant use of conventional DMARDs did not increase the likelihood of maintaining remission on a tapered biologic dose, as we also reported here [3]. The most innovative aspect of our study is precisely framed in this need to determine which characteristics of the disease and the patient increase the possibility of sustained dose reduction. To address potential confounding due to differences in baseline characteristics, we included covariates with clinical relevance or *p* < 0.20 in univariable analysis in our multivariable logistic regression models. When analyzing the entire study population, we found that the absence of tobacco exposure (OR 3.98), male sex (3.26) and first-line use of advanced therapies (OR 4.8) were the three factors that were independently associated with the tapering status. When sub-analyzing only the subjects under TNFis, the linked factors were disease duration (OR 1.09), no tobacco exposure (OR 3.3) and first-line therapy (OR 3.7). Therefore, as we see it, factors such as male sex, smoking, disease duration and first-line therapy are the same ones that (along with others) have been repeatedly associated with a greater or worse survival of biological drugs used in PsA [12,13,14,15,16,17,18]. This means that in many cases the factors associated with biologic drug persistence are the same ones that would allow us to better select patients who are candidates for dose reduction (potential responders to this strategy). An added novelty of our study is that we have demonstrated that this dose tapering is attainable in a population mostly treated with TNFibs, something barely referenced to date [3]. In a recent study by Uhrenholt et al. among 142 patients randomized to tapering (*n* = 95) or control (*n* = 47), successful tapering was achieved by 32% and 2%, respectively. Tapering group was the only statistically significant independent predictor for successful tapering (RR 14.0) [19]. Although their study did not find the same factors associated with dose reduction as our study, it is quite striking that the number of patients on drug tapering (*n*: 95) as well as those who finally achieved successful tapering (32%) was the same as that reported here. Therefore, a robust conclusion is that in both prospective and retrospective dose reduction studies, one in three patients manage to sustain that dose reduction, at least for those treated with TFNi.

We must acknowledge some drawbacks of our research. First, the retrospective, observational nature means admitting biases and confounders of various kinds that limit the quality of the results obtained, despite performing adjusted multivariate regressions. Second, the non-TNFi therapy arm was small and with a variety of therapies that does not allow us to ensure under which circumstances these other therapies could be subjected to dose reduction strategies. Third, the lack of a parallel, matched comparator group limits the causal interpretation of our findings; nonetheless, the use of adjusted multivariable analyses provides preliminary insights and generates hypotheses for future prospective research. However, we believe that our finding of one third of patients in successful tapering is consistent with the findings of much better designed and prospective studies, which gives robustness to our results (external validity). In addition, we have reported these tapering results in a population mostly treated with biosimilars, something barely reported to date [20], which contributes to boosting confidence in these therapies. Finally, although the high prevalence of TNFib use may limit the generalizability of our results to other treatment contexts, it also offers valuable real-world data on a scarcely studied population of PsA patients undergoing dose tapering.

## 5. Conclusions

To summarize, we have shown that after switching from a reference molecule to a biosimilar, most patients maintain the efficacy of the former. We have also shown that one in four patients are susceptible to a successful dose reduction strategy, and that this is possible with biosimilars, especially in patients who achieve a sustained therapeutic goal, and who in addition are male, non-smokers and on first-line treatment. Our findings suggest that TNFi biosimilars may be a feasible and potentially cost-effective option for dose tapering in well-selected PsA patients [21]; however, larger prospective studies with standardized protocols and longer follow-up are needed to confirm these results.

## Figures and Tables

**Table 1:** Disease characteristics of the study population.

Variable	N: 130
Age, years, mean (SD)	55.6 (11.2)
Male, *n* (%)	64 (49.2)
Female, *n* (%)	66 (51.8)
University degree, *n* (%)	28 (21.5)
Disease duration, years, median (IQR)	8.0 (3.0–13.0)
Smokers, *n* (%)	37 (28.5)
Former smokers, *n* (%)	29 (22.3)
Alcohol drinkers, *n* (%)	22 (17)
Weight, mean (SD)	77.7 (15.3)
CV comorbidity:	
Diabetes, *n* (%)	15 (11.5)
Hypertension, *n* (%)	39 (30)
Dyslipidemia, *n* (%)	49 (37.7)
Hyperuricemia, *n* (%)	38 (29.2)
CV events, *n* (%)	5 (3.8)
Family history:	
Psoriasis, *n* (%)	55 (42.3)
PsA, *n* (%)	12 (9.2)
CRP, median (IQR)	0.20 [0.0–4.70]
CRP ≥ 0.5 mg/dL, *n* (%)	31 (23.8)
PsA pattern:	
Peripheral, *n* (%)	97 (74.6)
Mixed, *n* (%)	26 (20)
Axial, *n* (%)	6 (4.6)
PsA features:	
Dactylitis, *n* (%)	53 (40.8)
Enthesitis, *n* (%)	37 (28.5)
Uveitis, *n* (%)	1 (0.8)
Nail disease, *n* (%)	34 (26)
Outcomes:	
DAPSA, median (IQR)	5.0 (0.4–10.0)
DAPSA remission, *n* (%)	57 (43.8)
DAPSA low, *n* (%)	55 (42.3)
Physician’s GDA, median (IQR)	2.0 (0.0–4.0)
Treatment:	
Methotrexate, *n* (%)	47 (36.2)
Leflunomide, *n* (%)	12 (9.2)
TNF inhibitors, *n* (%)	95 (73.1)
IL-17 inhibitors, *n* (%)	14 (10.8)
Ustekinumab, *n* (%)	2 (1.5)
Apremilast, *n* (%)	13 (10)
Tofacitinib, *n* (%)	6 (4.6)
Therapy line:	
1, *n* (%)	78 (60)
2, *n* (%)	24 (18.5)
≥3, *n* (%)	28 (21.5)
Median exposure to advanced therapies, years (IQR)	1.7 (0.9–4.7)
* Dose tapering, *n* (%)	32 (24.6)

N, *n*: numbers; SD: standard deviation; CV: cardiovascular; PsA: psoriatic arthritis; CRP: C-reactive protein; DAPSA: disease activity score for PsA; GDA: global disease assessment; TNF: tumor necrosis factor; IL: interleukin; IQR: interquartile range. CRP values are expressed in mg/dL; * 30 (31.6%) of the 95 patients treated with TNF inhibitors achieved sustained dose reduction, while this was only possible in 2 patients (5.7%) within the non-TNF inhibitor group, *p* = 0.002.

**Table 2:** Drug tapering-associated factors (*n*: 130). Full regression models.

Univariate Regression Model OR (95%CI), *p*-Value	Multivariate Regression Model OR (95%CI), *p*-Value
Male 2.8 (1.2–6.6), 0.018	**Male** **3.26 (1.26–9.04), 0.018**
Age 1.02 (0.98–1.052), 0.430	
Weight 0.99 (0.96–1.01), 0.347	
Disease duration 1.08 (1.02–1.15), 0.008	Disease duration 1.06 (0.99–1.13), 0.090
No tobacco exposure 3.94 (1.4–11.5), 0.012	**No tobacco exposure** **3.98 (1.30–14.20), 0.021**
Alcohol 1.23 (0.44–3.50), 0.696	
University degree 1.70 (0.68–4.28), 0.259	
Hypertension 0.75 (0.30–1.89), 0.539	
Diabetes 0.45 (0.10–2.12), 0.313	
Dyslipidemia 1.24 (0.55–2.83), 0.603	
Hyperuricemia 1.45 (0.61–3.41), 0.400	
Cardiovascular events 1.06 (0.11–10.50), 0.963	
Nail disease 0.63 (0.23–1.71), 0.365	
Enthesitis 0.65 (0.25–1.68), 0.374	
Dactylitis 1.05 (0.46–2.38), 0.912	
Axial disease 0.88 (0.32–2.42), 0.805	
C-reactive protein 0.88 (0.77–1.00), 0.058	
Physician’s global disease assessment 0.54 (0.40–0.73), <0.001	
DAPSA 0.73 (0.63–0.85), <0.001	
Methotrexate 1.62 (0.71–3.69), 0.249	
Leflunomide 1.67 (0.47–5.96), 0.432	
TNF inhibitors 15.2 (1.99–116.7), 0.009	
First-line therapy 4.8 (1.7–13.5), 0.003	**First-line therapy** **4.8 (1.7–16.7), 0.006**
Biosimilar 0.63 (0.13–3.02), 0.564	

OR: odds ratio; DAPSA: disease activity score for psoriatic arthritis; TNF: tumor necrosis factor.

**Table 3:** Dose tapering-associated factors among TNF inhibitor users (*n*: 95). Full regression models.

Univariate Regression Model OR (95%CI), *p*-Value	Multivariate Regression Model OR (95%CI), *p*-Value
Male 2.41 (0.96–6.04), 0.061	
Age 1.03 (0.99–1.07), 0.196	
Weight 0.98 (0.95–1.01), 0.189	
Disease duration 1.12 (1.04–1.20), 0.003	**Disease duration** **1.09 (1.01–1.18), 0.030**
No tobacco exposure 4.05 (1.32–12.50), 0.015	**No tobacco exposure** **4.06 (1.28–14.72), 0.023**
Alcohol 1.23 (0.41–3.71), 0.716	
University degree 1.56 (0.59–4.16), 0.373	
Hypertension 0.74 (0.27–2.0), 0.550	
Diabetes 0.25 (0.03–2.06), 0.196	
Dyslipidemia 1.22 (0.51–2.94), 0.653	
Hyperuricemia 0.98 (0.39–2.44), 0.961	
Cardiovascular events 2.21 (0.13–36.52), 0.580	
Nail disease 0.56 (0.20–1.59), 0.277	
Enthesitis 0.99 (0.36–2.77), 0.99	
Dactylitis 1.01 (0.42–2.42), 0.981	
Axial disease 1.10 (0.37–3.29), 0.859	
C-reactive protein 0.90 (0.77–1.05), 0.170	
Physician’s global disease assessment 0.57 (0.41–0.79), <0.001	
DAPSA 0.73 (0.62–0.87), <0.001	
Methotrexate 1.09 (0.46–2.59), 0.852	
Leflunomide 9.85 (1.05–92.32), 0.045	
First-line therapy 2.56 (0.86–7.60), 0.091	**First-line therapy** **3.70 (1.16–14.10), 0.037**
Biosimilar 0.63 (0.13–3.02), 0.564	

OR: odds ratio; DAPSA: disease activity score for psoriatic arthritis.

## Data Availability

The materials and raw data described in the manuscript will be freely available to any researcher without breaching any participant’s confidentiality. To facilitate the revision of the results by other researchers, a file with the patient data is available as an excel file upon request from the corresponding author.
